# Autologous and Allogenous Antibodies in Lung and Islet Cell Transplantation

**DOI:** 10.3389/fimmu.2016.00650

**Published:** 2016-12-23

**Authors:** Deepak Kumar Nayak, Prathab Balaji Saravanan, Sandhya Bansal, Bashoo Naziruddin, Thalachallour Mohanakumar

**Affiliations:** ^1^Norton Thoracic Institute, St. Joseph’s Hospital and Medical Center, Phoenix, AZ, USA; ^2^Baylor Research Institute, Dallas, TX, USA; ^3^Baylor Simmons Transplant Institute, Dallas, TX, USA

**Keywords:** lung, islet of Langerhans, transplantation, antibody, graft rejection

## Abstract

The field of organ transplantation has undoubtedly made great strides in recent years. Despite the advances in donor–recipient histocompatibility testing, improvement in transplantation procedures, and development of aggressive immunosuppressive regimens, graft-directed immune responses still pose a major problem to the long-term success of organ transplantation. Elicitation of immune responses detected as antibodies to mismatched donor antigens (alloantibodies) and tissue-restricted self-antigens (autoantibodies) are two major risk factors for the development of graft rejection that ultimately lead to graft failure. In this review, we describe current understanding on genesis and pathogenesis of antibodies in two important clinical scenarios: lung transplantation and transplantation of islet of Langerhans. It is evident that when compared to any other clinical solid organ or cellular transplant, lung and islet transplants are more susceptible to rejection by combination of allo- and autoimmune responses.

## Introduction

Solid organ transplantation is increasingly used as a clinical intervention to compensate for functional loss of an organ and to maintain metabolic homeostasis. This palliative treatment option can extend the lifespan and improve the quality of life for recipients, but outcomes vary depending on which organ is transplanted. Lung transplantation (LTx) can be a life-saving measure for patients with many severe chronic lung diseases, including chronic obstructive pulmonary disease, idiopathic pulmonary fibrosis, cystic fibrosis, alpha-1 antitrypsin deficiency, pulmonary hypertension, interstitial lung disease, and bronchiectasis. Unfortunately, LTx currently has the lowest long-term survival rate compared with survival rates associated with transplantation of other solid organs, its half-life (*t*_1/2_) being just 5 years. The primary reason for this low survival rate is chronic rejection, the clinical diagnosis for which is bronchiolitis obliterans syndrome (BOS).

Transplantation is not limited to solid organs. Allogeneic endocrine cell transplantation of islets of Langerhans is a treatment option for patients with autoimmune diabetes mellitus [i.e., type 1 diabetes (T1D)]. Transplantation of these cells is a replacement therapy that augments production of endogenous insulin. Islet cells are often isolated from multiple cadaveric donors and recipients require a continuous immunosuppressive therapy. However, long-term sustained normoglycemia is exceedingly difficult to achieve from islet transplantation; the mechanisms that lead to destruction of the islet allografts will be discussed in this review. Autologous islet transplantation—which also aims to restore endocrine pancreatic function after total pancreatectomy—is not as susceptible to rejection and therefore will not be discussed in this review. Because transplantation depends upon available donor organs and cells, genetic mismatches between donor and recipient become the focal points of the recipient’s immunologic responses. Resultant T cell and antibody (Ab) responses have been known to influence, often negatively, both short- and long-term functioning of transplanted allografts, and such incidences frequently portend the unfavorable consequences of graft loss (i.e., rejection).

In this review, we describe the spectrum of Ab responses observed in LTx and in transplantation of islets of Langerhans. LTx, whether unilateral or bilateral, involves surgical replacement of a diseased organ with normal and functioning lungs procured from a cadaveric donor. By contrast, allogeneic islet transplantation involves intrahepatic delivery of donor-isolated islet cells that supplement existing islet cells and insulin production resulting in normoglycemia. With recent increases in the prevalence of chronic obstructive pulmonary disease, cystic fibrosis, and T1D, the demand for transplantable lungs and pancreatic islets has increased. Since 2014, nearly 60,000 LTx procedures have been performed worldwide (both lungs-only and combined heart–lung transplantation; International Society for Heart and Lung Transplantation,^1^ accessed on July 25, 2016). As of 2012, approximately 1,400 islet cell transplantations have been recorded (Collaborative Islet Transplant Registry,^2^ accessed on July 25, 2016). Additionally, combined transplantation of lungs and pancreatic islets is an effective treatment option to restore respiratory and pancreatic insufficiency in terminal cystic fibrosis (1). The global need for organ transplantation is rising steadily with more prospective transplant recipients added to active waitlists (both first-time transplantation and re-transplantation due to graft failure). Concurrently, an acute shortage persists on the availability of transplantable organs (Organ Procurement and Transplantation Network^3^).

## Antibodies in LTx

Histocompatibility studies originated from the need to decipher mechanisms of graft rejection; these studies ultimately led to the identification of major histocompatibility complex (MHC) proteins. The human counterpart of MHC is the human leukocyte antigen (HLA) system. From an immunologic perspective, the revelation of HLA was critical to the understanding of immune responses—not only as they affect graft rejection, but also how they impact infectious disease, autoimmunity, and tumor biology. Notwithstanding the antigen presentation *via* peptide–MHC complexes that is central to T cell immune recognition and responses, MHC represents the bulk of steady state expression of surface proteins (up to 70,000 molecules per cell) (2). Class I MHC is ubiquitously expressed on every nucleated cell, whereas class II MHC is preferentially expressed on professional antigen-presenting cells (e.g., dendritic cells, macrophages, and B cells).

With more than 200 loci identified, the polygenic nature of HLA combined with high allelic polymorphism (>14,000 alleles for HLA class I and II combined,^4^ assessed on November 17, 2016) confers great diversity to HLA molecules (3–6). Furthermore, codominant expression of HLA allows for simultaneous expression of both paternal and maternal HLA haplotypes, which further increases the diversity of the HLA repertoire expressed in a given individual. Because of the high preponderance of HLA class I on every type of cell (i.e., ciliated, non-ciliated, and secretory epithelial cells; endothelial cells; basal cells; and connective tissue) and HLA class II on resident antigen-presenting cells (i.e., lung-resident macrophages and dendritic cells) and B cells, mismatched donor HLA molecules are easily recognized and quickly targeted by the recipient’s immune system after transplantation.

Although graft failure was long suspected to be a result of immunological complications, the host-adaptive immune response to MHC antigens wasn’t confirmed until 1956, when immunization of malignant cells in mice induced *de novo* Abs against MHC molecules (7). In a clinical setting, the association of preexisting HLA Abs with graft failure was witnessed when a large number of kidney transplant recipients who experienced acute graft rejection had donor HLA Abs (i.e., positive crossmatch), whereas recipients who lacked anti-HLA (i.e., negative crossmatch) had significantly higher graft survival (8, 9). Since these landmark studies, preexisting and *de novo* donor-specific antibodies (DSA) to mismatched HLA have generated a tremendous amount of clinical interest and have been widely applied in the study of all solid organ transplantation (10). The posttransplant development of *de novo* DSA was first documented following LTx in 2002 (11). Since then, a strong clinical association of *de novo* DSA with acute and chronic lung allograft rejection has been confirmed by many independent studies (12–20). Significantly, an association between the extents of donor–recipient HLA mismatches and incidence of chronic rejection (i.e., BOS) has been established (21) indicating a role for anti-HLA immune responses in the post-LTx acceptance and performance of lung allografts.

The pathogenicity of MHC Abs has been demonstrated in our laboratory using a mouse model of obliterative airway disease (OAD), in which ligation of MHC by antibodies led to OAD and lung-restricted autoimmunity (22, 23). In this model, exogenous delivery of anti-MHC class I or anti-MHC class II to the lung microenvironment induced small airway occlusion and fibrosis, creating pathologic lesions similar to those observed in humans with chronic lung graft rejection. While the Ab repertoire associated with lung graft rejection is not fully characterized, *de novo* anti-HLA class I and II titers, even when non-persistent, significantly predispose to chronic rejection (11, 15, 17, 19, 24–28). The alloimmune priming of HLA reactive B cells is believed to trigger loss of self-tolerance and development of cellular and humoral autoimmunity (26, 29). Owing to clinical significance, a number of transplant centers now routinely screen prospective LTx recipients for preexisting DSA for an immediate pretransplant desensitization and monitor for *de novo* DSA during post-transplant period.

In addition to HLA, several non-HLA molecules have been targeted by immune responses after allogeneic transplantation, which can influence post-LTx outcomes. Abs to MHC class I chain A (MICA) were reported to develop after DSA and were significantly correlated with BOS development (30). Abs to mismatched HLA or MICA are suspected to induce immune responses to various tissue-restricted self-antigens (26, 30). Development of Abs to filamentous self-proteins such as Collagen V (Col V) and K-alpha1 tubulin (Kα1T) have been studied in LTx recipients with great interest (31), and in experimental mice with OAD (22, 23). Col V forms the core component of the fibrillar extracellular matrix (ECM) in the lungs, and Kα1T is a cytoskeletal protein involved in intracellular locomotion. Preexisting anti-Col V and anti-Kα1T have also been associated with primary lung graft dysfunction (32, 33), which predisposes LTx recipients to development of both acute rejection (34) and BOS (35). Furthermore, role of anti-Col V and anti-Kα1T has been demonstrated in murine orthotopic LTx model where exogenous Ab administration disrupted an established lung graft tolerance resulting in fibrotic lesions in small airways and elicited lung-directed cellular autoimmunity (36). Despite the current focus on these two tissue-associated self-antigens (i.e., Col V and Kα1T) as a measure of lung-restricted autoimmunity, it is likely that a larger antigenic repertoire participates in lung graft-directed immune responses and rejection. Further analysis of these putative antigens may help delineate the pathogenic processes and facilitate development of new therapeutic strategies.

### Intricacy of B Cell Targets

Terasaki proposed a “humoral theory” to explain the basis of Abs influencing allograft rejection (37). This theory, formulated after in-depth analysis of kidney, heart, lung, and liver transplants, states that detection of graft-specific Abs is a reliable measure of humoral sensitization and an early indicator of graft rejection. The humoral theory gained credence when anti-donor humoral response was established to be the major factor in hyperacute and chronic graft rejection (10, 38). B cell sensitization against mismatched donor HLA may readily occur as they are non-self proteins and, by virtue of their cell-surface expression, are amenable to B cells. Further, an indirect antigen presentation pathway has been established in which a recipient’s antigen-presenting cells acquire the donor antigens, activating antigen-specific CD4 T cells that provide necessary costimulation for B cell priming (39). An intercellular antigen transfer has also been described in LTx, wherein recipient’s antigen-presenting cells acquire and cross-present donor antigens *via* a “semidirect” pathway (40).

The generation of Ab to sub-surface non-HLA antigens (including various tissue-restricted self-antigens) is poorly understood. Col V is an important component of heterotopic collagen fibers, as it initiates the fibril assembly by serving as a nucleator to Collagen I and regulates the number and length of fibrils (41). Col V is a minor component (constituting nearly 2–5% of total collagen in most tissue) and remains buried in the healthy collagen fibers, while Kα1T is a polymerized cytoskeletal protein of the microtubule. Given their intracellular sequestration, it is intriguing that Col V and Kα1T become targets and driving forces for immunopathogenesis of lung allograft rejection. In order for this self-antigen-directed reactivity to proceed, two significant immunologic requisites must be fulfilled: (1) Col V and Kα1T must be available to the immune system since B cell receptors can recognize native or linear epitopes, and the buried or “cryptic” antigens (i.e., Col V and Kα1T) must be accessible to the circulating B cells during post-LTx sensitization; and (2) the repertoire of Col V and Kα1T specific B cells must be intact and functional.

Mechanisms and modality of how the sequestered lung-restricted antigens may become bioavailable has generated significant clinical interests. Metalloproteases produced during transplant-related ischemic reperfusion injury to the lungs structurally impair fiber integrity and strip the collagen fibers, thereby exposing the core Col V fibers (42, 43). In addition, fragments of Col V are released into bronchoalveolar lavage fluid after allogeneic LTx (44). It is possible that during this immunologic assault (which may result in cell death), cytoskeletal components may become available for B cell priming. Nevertheless, the notion of a circulating pool of Col V and Kα1T reactive B cells requires thoughtful analysis and experimentation. Notwithstanding the ubiquitous expression of Col V and Kα1T, lack of a deletional tolerance may introduce risk of autoimmunity. The possible escape of Col V- and Kα1T-reactive B cells may be due to an incomplete clonal deletion, or the immune response directed toward altered/neo-epitopes on Col V and Kα1T generated by posttranscriptional modifications. Recent study strongly suggests that the breakdown of peripheral tolerance *via* T regulatory cell populations may affect the development of immune responses to these fibrillar proteins (45). Nonetheless, clonal tolerance has been successfully achieved in a rat LTx model, in which oral administration of Col V induced protection from chronic rejection (46, 47).

While several possible routes may exist by which donor antigens become available to immune priming, our laboratory and others’ have recently demonstrated a long-term persistence of donor-derived alveolar macrophage (AM) in transplanted lungs (48, 49). The donor AMs act as a reservoir of donor HLA and are available for stimulating graft-infiltrating B cells. We have also shown that mismatched MHC present on AM is sufficient to elicit anti-donor T cells and Abs. Therefore, the donor AM may initiate and/or contribute to the post-LTx DSA responses. At the instance of a matched HLA class II allele between donor and recipient, donor AMs can participate in direct presentation of endogenous antigens (donor-derived) to the recipient’s CD4 T cells.

In order to define the spread of alloantigenic immune responses in to tissue-restricted autoimmune response, we recently characterized exosomes isolated from serum samples from LTx recipients (50). Exosomes are membrane-bound nano-vesicles involved in cell-to-cell communication. In our study, exosomes from patients with acute rejection and with BOS contained donor HLA, Col V and Kα1T, and various immunostimulatory microRNAs. Exosomes from stable LTx recipients, however, contained neither Col V nor Kα1T and featured a profile of immunoregulatoy microRNAs. It is hypothesized that exosomes in patients with graft rejection are immunogenic and that these exosomes can essentially traffic and deliver their antigenic cargo toward priming of allogeneic donor HLA and lung-restricted autoantigen specific of CD4 T cells (50). The role of exosomes has also been recognized in other solid organ transplantations (51). Whether or not exosomes participate in the antigen presentation by semidirect pathway has not been established, and their contribution to the indirect and direct pathways of antigen presentations remains to be validated.

Analysis of ECM, as a potential source of lung-restricted self-antigens, in small airway inflammation and fibrosis associated with BOS is unconventional. Biochemically, ECM is a complex adduct of glycoproteins, collagens, and polysaccharides and is responsible for homeostatic maintenance of the lungs, including their development, maturation, and post-injury tissue repair (52, 53). In patients with fibrotic lung diseases or who develop BOS after LTx, aberrant ECM deposition leads to permanent tissue scarring. An alloimmune reaction directed at the lung graft generates “redox hotspots” with surplus reactive oxygen species. Redox reactions in the lungs are known to modulate signaling and composition of ECM (54), pericellular localization, and extracellular focal plaque formation (55). The inflammatory nature of ECM, particularly with regards to ECM-infiltrating immune cells and composition of the fibrotic scars, may indicate the role of ECM as an antigen reservoir and initiator of inflammation that prompts lung-directed autoimmune responses.

With regards to small airway epithelial cells, club cells may play an important role in the elicitation and/or amplification of lung graft-directed immune responses. Club cell secretory protein (CCSP) is an important component of the pulmonary surfactant that has an anti-inflammatory function (56–60). A significant reduction in bronchoalveolar CCSP and club cells has been reported in LTx recipients who develop BOS compared to stable LTx recipients (61). These results suggest that declining CCSP may augment both the innate and adaptive immune responses that lead to lung allograft rejection.

### Pathogenesis

The tenets of Terasaki’s humoral theory (10, 37, 38) on the primary role of HLA Abs in solid organ rejection remain generally undisputed, and the development of Abs and their correlation with lung graft rejection has been increasingly reported across transplant centers. Because adaptive cellular and humoral effectors work in tandem in the elicitation of graft-directed immune responses, it is difficult to ascribe a dominant role for one over the other. With respect to immunologic factors associated with allograft rejection, Terasaki and Cai suggested that: (1) Abs play a causative role in the pathogenesis of graft rejection and (2) acute cellular rejection (ACR) can be of humoral origin (38).

Antibody-mediated rejection (AMR) of a lung graft results in three primary, interdependent manifestations: (1) hyperacute rejection, (2) acute humoral rejection, and (3) chronic lung allograft dysfunction (CLAD). Although AMR lesions are well defined in renal and cardiac allografts, the criteria for assessment of lung AMR are continuously changing based on varying immunopathology observed in pulmonary biopsies. The recent International Society for Heart and Lung Transplantation grading criteria for evaluating lung graft rejection define pulmonary AMR as presence of donor-HLA-specific Abs and characteristic lung histology that may or may not be accompanied by complement deposition in the graft (62). Furthermore, AMR may persist subclinically—that is, without being detected. Occurrence of preformed, sometimes-low-titer Abs to donor-HLA, Col V or Kα1T pre-LTx have been shown to increase risk of graft rejection (33, 63). Delivery of exogenous anti-Col V and anti-Kα1T produced AMR in experimental murine LTx (32, 64). ACR is a common but reversible immune reaction, with characteristic perivascular or peribronchiolar mononuclear infiltrations. AMR, on the other hand, is difficult to diagnose and may accompany local activation of complements caused by DSA (65–67).

Higher frequency and high grade of AMR are risk factors for development of HLA Abs and BOS (34, 68), but AMR exhibits a strong association with *de novo* development of anti-HLA, significantly increasing the risk of BOS (24). BOS continues to be the major cause of posttransplant morbidity and mortality, affecting approximately 50% of patients with transplanted lungs within 5 years of LTx (69). It clinically manifests with progressive, irreversible loss of respiratory function (>20% of baseline) that is unresponsive to any immunosuppressive regimen (70). A large body of work has established a strong humoral link that predisposes for development of BOS after LTx. DSA directed to MHC class I and II proteins, even when detected only transiently, poses significant and independent risks for BOS development and influences its onset kinetics, severity, and mortality (11, 15, 17, 19, 24–28, 71).

In addition to BOS, restrictive allograft syndrome (RAS) has been recently described as another form of CLAD after human LTx (72). RAS is an airway-restrictive phenotype and is more aggressive than BOS, with median survival of just 6–18 months after diagnosis (73–76). The alloimmune priming of donor-HLA reactive B cells is believed to trigger loss of self-tolerance and intermolecular epitope spreading, eliciting cellular and humoral responses directed to Col V and Kα1T. Therefore, a functional interplay has been proposed in which donor-directed alloimmunity leads to development of tissue-restricted autoimmunity to self-antigens (26, 29, 77).

The immunodominant role of DSA in chronic rejection has been recognized by three distinct observations: (1) *de novo* DSA is associated with recurrent and high-grade cellular rejection and lymphocytic bronchiolitis (11, 15), (2) development of DSA often precedes *de novo* Col V- and Kα1T-specific Abs (26), and (3) depletion of the circulating Abs by Ab-directed therapy offers protection from BOS with a lower hazard ratio and enhanced pulmonary function (25, 27, 78–80). Preexisting Abs to Col V and Kα1T have been found in different terminal lung diseases pre-LTx, and such pretransplant autoantibodies were significantly correlated with poor outcomes, including development of DSA and BOS (33, 81). Moreover, the absence of preexisting Abs to lung self-antigens was correlated with freedom from *de novo* MHC class I and class II DSA and with lower incidence of BOS (33). Preformed antibodies to lung-restricted self-antigens without measurable DSA have also been associated with BOS development after human LTx (24). In summary, the current consensus is that both DSA and Abs to lung-restricted self-antigens (whether preformed or *de novo*) are significant risk factors for all forms of lung allograft rejection and limit both short- and long-term success of LTx.

### Diagnosis

Sensitive detection and measurement of graft-specific Abs have been closely linked with the evolution of allogeneic organ transplantation, often serving as a designation of success in solid organ transplantation. With outcomes being dependent on the extent of antigenic mismatches between donor and recipient, optimization of matching strategies has been the subject of much discussion. A consensus guideline has been formulated for Ab testing in transplantation (82). Crossmatching was an early test in which recipient’s serum was mixed with donor’s cells to detect presence of anti-donor Abs. A negative crossmatch—first applied in renal transplantation—was effective in minimizing graft failure (9). The development of the complement-dependent cytotoxicity test utilized complement fixation and mixing of donor lymphocytes with recipient’s serum in the presence of complement. This assay was further refined when antiglobulin was added to augment the reaction (83).

More recently, however, flow cytometry has been the test of choice to define crossmatch compatibility, given its high sensitivity. A shortcoming of crossmatching is the amount of time it takes—it was difficult to perform prospectively and it increased cold ischemic time of the organ. Currently, a virtual crossmatch is preferred, especially for lung and heart transplantation procedures. The development of the “HLA/Teraski plate” was a crucial step in the commercialization of assay reagents, as it enabled testing for presence and specificity of panel-reactive antibodies (PRAs). Recently, solid-phase immune assays such as FlowPRA (OneLambda, Canoga Park, CA, USA), LABScreen (OneLambda, Canoga Park, CA, USA) and Lifecodes LifeScreen (Immucor, Peachtree Corners, GA, USA) were developed. FlowPRA is a flow cytometry-based immunoassay for rapid detection of HLA class I and class II Abs in human serum. Using a unique set of recombinant HLA protein adsorbed fluorescent microbeads binding of specific Abs in serum is detected by flow cytometry. LABScreen and LifeScreen, on the other hand, allow precise determination of allelic HLA and/or MICA on a Luminex (Luminex Corporation, Austin, TX, USA) platform. Currently, assays such as LABscreen and FlowPRA are standard and are routinely conducted for testing of PRAs at HLA laboratories. These assays can successfully assign antigenic specificities to perform virtual crossmatching.

In stark contrast to the advancements in immunologic detection of HLA Abs, progression of methods to identify and characterize Abs to tissue-restricted self-antigens is in its infancy. The diversity of tissue-restricted self-antigens involved in allograft rejection is still being investigated, and there are no validated kits commercially available for detecting immune responses to self-antigens. In addition, a possibility of low titer circulating Abs (below detection limit) as the graft undergoing rejection may already sequester them further complicates the detection issue. A solid-phase protein microarray has been employed to screen targets of humoral autoimmunity following LTx (84). Given the nature of nuanced B cell antigenic determinants (i.e., conformational vs linear; continuous vs discontinuous), cellular localization (i.e., surface-bound, cytosolic, or nuclear), and posttranscriptional modifications, the results obtained from solid-phase assays are limited in their capacity to gauge the spread of humoral autoimmunity. This prevents full realization of the Ab repertoire and limits researchers’ ability to identify novel targets. Currently, home-grown enzyme-linked immunosorbent assays are the most commonly used procedures for detecting Abs to various tissue-restricted self-antigens (31, 32).

## Clinical Pancreatic Islet Transplantation

Transplantation of whole pancreas or isolated pancreatic islets are two effective treatment options for brittle (i.e., severe) T1D patients, as both procedures replace the depleted β-cell mass lost due to autoimmunity. Islet transplantation has the advantage of being a minimally invasive procedure compared to transplantation of pancreas. Most of T1D patients receive exogenous insulin therapy to control blood glucose levels, but this often results in severe and recurrent of hypoglycemia (85). Many patients who undergo islet transplantation achieve normoglycemia and experience freedom from the life-threatening consequences of severe hypoglycemic episodes. Despite significant advances in the islet isolation protocol, islets isolated from more than one donor pancreas are often required to achieve insulin-independent status (86), and a majority of islet transplant recipients return to some form of exogenous insulin usage within a few years of transplantation due to chronic rejection (87).

According to the Collaborative Islet Transplant Registry (See text footnote 2) data collected from the islet transplant centers around the world indicate 1- and 5-year insulin-independence rates after final islet infusion at 50% and 23%, respectively. Majority of the procedures performed in North America are islet transplant alone where as a higher number of procedures performed at European centers are simultaneous islet-kidney allograft (SIK) transplantation or islet after kidney allograft (IAK) transplantation. The SIK/IAK rate in North America is 10% were as that in European centers are >35%. A small number of islets after LTx have been successfully performed at the GRAGIL (Groupe Rhin-Rhône-Alpes-Genève pour la Transplantation d’Ilots de Langerhans) consortium (1). This combined transplantation was particularly beneficial for patients with end-stage cystic fibrosis and severe cystic fibrosis-related diabetes. Long-term follow-up on transplant recipients resulted in persistent improvement of glycemic control with normalized glycated hemoglobin (HbA_1c_) in conjunction with significant (50%) reduction in the daily exogenous insulin requirement. The normoglycemia was durable and was preserved for a durable period of 15 years in these lung-islet combined transplant recipients.

Multiple factors contribute to loss of islet grafts, including poor islet quality, posttransplant inflammation, immunosuppressive drug-induced toxicity, recurrent autoimmunity, β-cell exhaustion, and alloimmune responses (88). Primary causes of islet allograft rejection are thought to be incidences of β-cell directed autoimmunity in combination with the alloimmune response to multiple mismatched HLA antigens that significantly impact long-term islet cell function.

### Alloimmunity against Islet Grafts

Introduction of allogeneic tissue into the body during solid organ or cellular transplantation is known to produce *de novo* anti-HLA, which plays a major role in acute and/or chronic graft failure. Rejection due to alloimmunity after islet transplantation is mainly due to the Abs against donor-specific HLA class I and II molecules (89, 90). Moreover, the requirement of multiple islet infusions to achieve insulin independence exposes transplant recipients to an unusually high number of HLA mismatches, resulting in elevated risk for a broader spectrum of donor-specific HLA Abs (88). Our group researched a possible role for HLA Abs in the rejection of islet allografts (91). This report was followed by a comprehensive analysis of both cellular and humoral responses against donor-specific antigens in seven islet transplant recipients, and a clear association of flow cytometry-detected and immunospot-detected T cells with islet graft failure was revealed (92). Of these seven patients, three with positive donor-specific responses rejected islet allografts, either acutely or chronically. A concurrent report by Rickels et al. reported islet graft failure in two of six patients with detectable HLA class I and II antibodies (93). Cardani et al. analyzed HLA sensitization in 66 patients who underwent islet transplantation at a single center between 1985 and 2006 and reported no significant correlation between positive PRA and islet graft transplantation outcome (94). However, loss of islet graft function was associated with positive PRA after immunosuppression tapering or infection. The study by Cardani et al. also revealed that 24% patients developed PRA after immunosuppression was discontinued.

The Edmonton group revealed its findings on the role of anti-donor HLA in the islet allograft rejection in a large patient cohort, screening posttransplant HLA Abs in 98 patients. In their cohort, 29 patients (30%) developed DSA, including 23 recipients (23.5%) who developed DSA while still on immunosuppression. Ten of the fourteen patients (71%) who discontinued immunosuppression developed extensive amounts of PRA. This report documented posttransplant HLA sensitization among patients who had negative PRA prior to transplantation. Importantly, the fasting C-peptide, which is an indicator of islet graft function, was significantly lower in sensitized recipients compared to non-sensitized recipients (95). Another study showed that pretransplant detection of PRA to MHC class I and II (>15%) among recipients was associated with increased need for insulin post-transplant (96).

The Collaborative Islet Transplant Registry offered a comprehensive analysis of 308 patients who received islet transplantation at different centers between 1999 and 2008. They found that HLA class I sensitization both pre- and posttransplant was correlated with islet graft failure. Unlike any other type of organ transplantation, allogeneic islet recipients could be exposed to a total of 9–25 HLA class I and II antigen mismatches. This extraordinarily high number of mismatches was likely due to the multiple HLA-mismatched pancreas donors used for islet isolation, and also possibly the result of increased risk of patients receiving any future transplants (88). A significant increase in the level of HLA Abs was seen, even when patients adhered to an immunosuppression regimen. It would be beneficial if islet transplants could be performed with single-donor islet infusion, as it would minimize the risk of broad sensitization.

Despite clinical studies reporting a possible association between HLA sensitization and islet graft failure, causality has still not been definitively established. Previous *in vivo* studies have indicated an association between DSA and islet transplantation survival. Using a congenic rat islet transplant model, Bittscheidt and colleagues have reported that graft survival was significantly influenced by the degree of donor-recipient MHC matching as well as recipient presensitization (97).

Other reports have shown that the presence of pretransplant autoreactive T cells and *de novo* donor-specific cytotoxic CD4 T cells resulted in poor outcomes after islet transplantation (92, 98, 99). In fact, the preformed auto- and alloreactive Abs were considered to be negative indicators for survival of the islet graft (96, 98, 100). Thus, the adaptive immune system plays essential role in long-term islet graft survival and influences clinical outcomes. The allosensitization triggered by donor antigens likely elicits the interferon-γ- and interleukin (IL)-2-mediated T helper1 response, which is destructive for the function of the transplanted islets (92). An *in vitro* study of mixed islet leukocyte reaction by Bouwman et al. showed induction of direct T cell response with augmented responses in cases with two or more mismatches of HLA class II (101).

A recent contrasting report showed that 11/18 islet transplant recipients (61.1%) had preexisting anti-HLA, including 6 patients (33%) who developed *de novo* DSA against the HLA of the transplanted islets (90). Remarkably, no significant association existed between the newly developed DSA after islet transplantation and clinical characteristics such as recipient gender, age, number of post-transplant infections, HLA class I/II eplet mismatch, and immunosuppression protocol. Furthermore, the *de novo* DSA was not associated with reduced graft survival or function. Interestingly, the newly formed posttransplant anti-HLA class II Abs were related to the Predicted Indirectly Recognizable HLA Epitopes (PIRCHE-II^5^), specific to the T helper epitopes.

Long-term survival of an allogeneic islet transplant faces a greater challenge than solid organ or other cellular transplants due to the preexisting islet-specific autoimmunity and the likely scenario of multiple HLA-mismatched islet infusions. In a recent analysis of 59 consecutive islet transplant recipients, 39 (66%) experienced *de novo* titer increase in both DSA and autoantibodies (96). Patients with increased Ab titers had significantly lower graft survival. Furthermore, newly developed DSA was associated with MHC class II (HLA-DR) mismatches and preexisting PRA (96).

The expression of MHC class II in the endocrine tissue of freshly isolated pancreatic islets is nearly undetectable. However, posttransplant infection or the presence of proinflammatory cytokines can induce the expression of MHC class II. Such induced expression of MHC class II has been demonstrated *in vitro* and has been associated with development of donor-specific HLA class II Ab production after islet transplantation (91).

### Autoimmunity against Islet Grafts

Because pancreatic islet transplantation is often performed in patients with T1D, the existence of preformed autoantibodies against major islet-specific antigens is an inherent issue for most recipients. Autoantibodies in the circulation are formed during the pathogenesis of T1D prior to islet transplantation. Autoantibodies against pancreatic islets that are deemed clinically significant include anti-glutamate decarboxylase 65 (GAD65), anti-insulin autoantibody, anti-zinc transporter ZnT8, anti-islet cell autoantibody, and anti-tyrosine phosphatase autoantibody (IA-2) (102). Some of the earlier reports of posttransplant immune responses in islet transplant recipients suggested that no correlation existed between preexisting autoantibodies and islet graft failure (96, 98). Similar claims were made about the role of autoantibodies in clinical outcomes of whole pancreas transplants (103). However, preformed autoantibodies in islet recipients have indeed been associated with graft failure compared with recipients who did not have autoantibodies (104). Compared to preformed autoantibodies, the elevated recurrences of autoantibodies specific to ZnT8A, GAD65, and IA-2 in the posttransplant period were predictive of graft failure in islet transplantation (96) and foretold an 80% chance of graft failure in transplanted whole pancreas (105). In spite of the early notion of a non-association between preexisting autoantibodies and graft loss, analysis of these autoantibodies may be clinically useful in helping health practitioners anticipate recurrences of autoimmune graft rejection. A number of factors may affect reappearance of autoantibody, for example, suboptimal immunosuppression protocols that include CD25 antagonism or inhibition of mechanistic target of rapamycin (mTOR) by rapamycin. Antithymocyte globulin (ATG) and mycophenolate mofetil (MMF), meanwhile, decrease the risk of autoantibody recurrence (96).

An early study considered seven islet allograft recipients to determine the significance of autoreactivity in islet transplant outcomes (106). In this study, three out of seven recipients under ATG induction immunosuppression retained comprehensive islet function more than 1 year post-transplant, with less autoreactivity and with no alloreactivities. The remaining four patients received no ATG in their immunosuppression treatment. Of these, three patients lost islet function within 3 weeks, and one patient demonstrated hyper-autoreactivity without alloreactivity and experienced a delayed loss of islet function around 33 weeks, with recurrence of autoimmunity (106). This group later studied the incidence of allo- and autoimmunity in 29 islet transplant recipients 1 year after islet transplantation. Repeat analysis included modification in the immunosuppression regimen. ATG was a part of induction, and tacrolimus with MMF were included in the maintenance immunosuppression protocol. The outcomes of this study demonstrated that pre- and posttransplant autoimmunity were associated with delayed insulin non-requirement and autoimmunity was directly proportional to the recipients’ circulating C-peptide 1 year post-transplant. Moreover, seven out of eight recipients who had no history of pretransplant autoreactivity achieved insulin independence while none of the four recipients who had preformed autoreactivity, predominantly against GAD and IA-2, achieved insulin independence (107). These analyses suggest that including ATG in the induction immunosuppression may help control autoreactivity and improve islet graft function.

The association between a recipient’s pre- and posttransplant autoreactivity and clinical outcomes are often highly variable from center to center. A previous report (106) asserted that no correlation exists between recipients’ autoimmunity and graft function posttransplant, but others have stated that 60% of islet transplantation recipients with fewer autoreactive circulating GAD65 T cells achieved long-term insulin independence, whereas the 40% of patients with elevated levels of autoreactive GAD65-specific T cells producing proinflammatory cytokines and did not achieve long-term insulin independence (108). The findings of Chujo et al. are supported by another report, in which pretransplant GAD65 and IA-2 autoreactive T cells affected the 1-year insulin-independence rate of alloislet transplant recipients (107).

Allogeneic graft rejection and autoimmune recurrence make a critical contribution to long-term outcomes after islet transplantation, and CD4+ and CD8+ T cells are vital in the pathogenesis of graft rejection. Moreover, T1D recipients have an increase in autoreactive memory T cells (109). Donor-specific MHC molecules likewise play an important role in activation of immunogenic T cells in the recipients, affecting islet graft outcomes. Huurman et al. analyzed islet transplantation recipients’ cellular responses to donor-specific MHC class II antigens and measured the expansion of alloreactive CD4+ T cells by ^3^H-thymidine incorporation (110). They also measured release of pro- and anti-inflammatory cytokines in the culture supernatant *in vitro*. Recipients who achieved long-term insulin independence expressed greater IL-10 release and regulatory T cells compared to recipients with failed allografts, who showed more IL-2 release in the supernatant (110).

Autoreactive CD8+ T cells are thought to play an active role in the destruction of alloislet graft. Memory autoreactive T cells have a longer half-life, are preserved in the circulation for a long time and expand after exposure to the specific autoantigens posttransplantation. Velthuis et al. analyzed frequency of CD8+ T cells in T1D patients and islet transplant recipients and demonstrated that T cells reacting to insulin and pre-proinsulin epitopes increased after transplantation (111). Further, the presence of some alloreactive CD8+ T cells possibly due to donor/recipient MHC mismatch induced expansion of cytotoxic T cells causing acute islet graft rejection (99).

### Immunosuppression in Islet Transplantation

Understanding the role of islet-specific alloimmune responses and autoimmunity in islet graft function has been useful in designing effective immunosuppression regimens that can control graft failure. Most immunosuppression drugs commonly used in whole organ transplantation are either lethal to transplanted islets or induce diabetes in the recipients. For recipients of islet transplantations, azathioprine, cyclosporine, and corticosteroids are usually included in the immunosuppression regimen in the immediate posttransplant period. Most islet transplants are performed after renal or simultaneously with renal transplantation. Application of these immunosuppression regimens has produced variable posttransplant outcomes in islet transplantation (112–114). Cyclosporine sparing immunosuppression is preferred based on several *in vitro* studies that have documented its β-cell toxicity (112, 115, 116).

The outstanding posttransplant result achieved with the Edmonton protocol (i.e., seven out of seven patients became insulin-independent) was partly due to a modified immunosuppression regimen, which included no steroids, and included induction with daclizumab and maintenance with low-dose tacrolimus and sirolimus (117). Impaired graft function and β-cell proliferation were reported with sirolimus (118, 119); however, no clinically significant adverse effects or decrease in islet engraftment were reported (120). In spite of its diabetogenic property, inclusion of low-dose tacrolimus in the immunosuppression maintenance protocol effectively improved short-term islet graft function (121). For long-term islet graft function, however, the Edmonton protocol was not effective. Among 47 patients who achieved insulin independence, only four (8.5%) retained the insulin-independent status at 5 years (122).

Recent improvement in the immunosuppression protocol has significantly enhanced the clinical islet transplant outcomes as illustrated in Table 1. Long-term islet transplant outcomes were improved with the use of humanized anti-CD3 Ab (OKT3γ1 Ala-Ala) for induction that depletes mature T cells followed by administration of calcineurin and mTOR inhibitors. The same group later effectively used T cell depletion with ATG along with antitumor necrosis factor α in the induction regimen and achieved superior long-term insulin independence in 50% (4/8) of the recipients (123, 124).

**Table 1:** **Immunosuppression strategies in clinical pancreatic islet transplantation**.

Induction IS	Maintenance IS	No. of recipients	Type of transplant	II achieved	Year/reference
ATG + Bela	Sir + MMF	5	ITA	5	2010/(125)
ATG + Efa/	Sir + MMF	5	ITA	5	2010/(125)
ATG + ETA + Ana/	Tac/MMF	3	ITA	3	2011/(126)
Dac/	Tac/Sir	3	ITA	3	2011/(126)
ATG + Tep or ATG + TCDAb + TNFi	Tac or CsA/Sir or CsA/Sir or Eve	29	ITA	15	2012/(124)
TCDAb + TNFi	Tac or CsA/Sir or Eve	20	ITA	10	2012/(124)
TCDAb	Tac or CsA/Sir or Eve	43	ITA	N/A	2012/(124)
Dac	Tac or CsA/Sir or Eve	177	ITA	35	2012/(124)
ATG	Sir	12	ITA	5	2014/(127)
ATG	Tac + MMF	48	ITA	N/A	2014/(128)
ATG or Dac or Bas	Tac or Sir	38	SIK/IAK	4	2015/(129)
ATG or Bas	Sir + Tac	48	ITA	25	2016/(85)
ATG or Bas	Ste or Tac + Aza	18	ITA/SIK/IAL/SILL	9	2016/(90)

*Ale, alemtuzumab; Ana, anakinra; ATG, antithymocyte globulin; Aza, azathioprine; Bas, basiliximab; Bela, belatacept; CsA, cyclosporine A; Dac, daclizumab; Efa, efalizumab; Eta, etanercept; Eve, everolimus; Exe, exenatide; IAK, islet after kidney transplantation; IAL, islet after lung or liver transplantation; II, insulin independence achieved in no. of patients; Inf, infliximab; IS, immunosuppression; ITA, islet transplantation alone; MMF, mycophenolate mofetil; N/A, not available; SIK, simultaneous islet-kidney transplantation; SILL, simultaneous islet-liver-lung transplantation; Sir, sirolimus; Ste, steroids; Tac, tacrolimus; TCDAb, T cell depleting antibodies; TNFi, tumor necrosis factor-α inhibition*.

The Clinical Islet Transplantation Consortium^6^ recently reported encouraging results in 48 patients who underwent islet transplantation. All 48 T1D patients experienced severe hypoglycemic episodes and impaired awareness of hypoglycemia pretransplant, and all maintained a regimen of ATG or basiliximab for induction and sirolimus with low-dose tacrolimus for immunosuppressive maintenance (85). In this study, the primary end point fixed was the accomplishment of HbA_1c_ < 7.0% (53 mmol/mol) at 1 year and freedom from severe hypoglycemic episodes from day 28 to day 365 after initial transplant. The primary end point was effectively met by 42/48 patients (87.5%) at one and by 34/48 patients (71%) at two posttransplant years. Hypoglycemia awareness was significantly restored (*p* > 0.0001), with marked improvement in Clarke and HYPO scores in all patients in the study (85).

A major obstacle to successful islet engraftment is the activation of an innate immune response, called “instant blood-mediated inflammatory response” (IBMIR). This reaction is characterized by release of proinflammatory cytokines, infiltration of innate immune cells, and activation of coagulation pathways (130). IBMIR has been documented in all three forms of islet transplantation (i.e., autologous, allogenous, and xenogenous). It is estimated that roughly 50% of transplanted islets are irreversibly damaged during the peri-transplant period, usually within hours to days. Following islet transplantation, release of proinflammatory cytokines and chemokines has been reported (131). These soluble molecules include tissue factor, high-mobility group protein B1, cytokines and chemokines such as chemokine (C-C motif) ligand 2, chemokine (C-X-C motif) ligand (CXCL) 12, tumor necrosis factor (TNF) α, IL-1β, IL-6, and CXCL8/IL-8. Introduction of two anti-inflammatory compounds that inhibit TNF-α (etanercept), IL-1β (anakinra) has been shown to improve islet allograft function (126). However, a direct link between the control of IBMIR and development autoimmune and alloimmune responses has not been established in allogenic islet transplantation and will undoubtedly be the focus of future studies.

## Conclusion

In this review, we have synthesized the published reports on immune responses to alloantigens encoded by HLA loci and non-HLA tissue-restricted self-antigens in the pathogenesis of graft rejection after human lung and islet transplantation. Much work has been done to determine the role of HLA Abs in allograft rejection, but consideration of immune responses to non-HLA antigens (including tissue-restricted self-antigens) is still a new territory. Many recent studies have suggested that tissue-restricted self-antigens direct immune responses and play a meaningful role in allograft rejection. Nevertheless, it remains unclear how these tissue-restricted immune responses initiate and perpetuate graft rejection, how tolerance to these tissue-restricted self-antigens are broken, and what role alloimmunity plays in the pathogenesis of chronic rejection despite immunosuppressive regimens.

Current work would suggest a crosstalk between allo- and autoimmunity in both lung and islet transplantation. It is possible that, once initiated, immune responses to tissue-restricted self-antigens are not suppressed by the immunosuppressive regimen, and this smoldering immune reaction contributes to the onset and progression of chronic allograft dysfunction. In experimental models, there is evidence that Abs to both MHC and to tissue-restricted self-antigens can break established tolerance, suggesting that Abs are the driving force in the induction of chronic allograft dysfunction. Therefore, there is an urgent need to develop new diagnostic and/or therapeutic approaches to prevent Abs and to treat chronic allograft dysfunction.

## Author Contributions

DKN, PBS, BN, and TM conceptualized the review; DKN and PBS wrote the manuscript; SB participated in discussion; BN and TM critically revised the manuscript.

## Conflict of Interest Statement

The authors declare that the research was conducted in the absence of any commercial or financial relationships that could be construed as a potential conflict of interest.

## Footnotes

^1^www.ishlt.org/registries/.

^2^www.citregistry.org/.

^3^https://optn.transplant.hrsa.gov.

^4^https://www.ebi.ac.uk/ipd/imgt/hla/stats.html.

^5^http://company.pirche.org/.

^6^www.citisletstudy.org.
